# Long short-term memory RNN for biomedical named entity recognition

**DOI:** 10.1186/s12859-017-1868-5

**Published:** 2017-10-30

**Authors:** Chen Lyu, Bo Chen, Yafeng Ren, Donghong Ji

**Affiliations:** 1 School of Computer Science, Wuhan University, Wuhan, 430072 Hubei China; 2 Department of Chinese Language & Literature, Hubei University of Art & Science, Xiangyang, 24105 Hubei China; 3 Guangdong Collaborative Innovation Center for Language Research & Services, Guangdong University of Foreign Studies, Guangzhou, 510420 Guangdong China

**Keywords:** Biomedical named entity recognition, Word embeddings, Character representation, Recurrent neural network, LSTM

## Abstract

**Background:**

Biomedical named entity recognition(BNER) is a crucial initial step of information extraction in biomedical domain. The task is typically modeled as a sequence labeling problem. Various machine learning algorithms, such as Conditional Random Fields (CRFs), have been successfully used for this task. However, these state-of-the-art BNER systems largely depend on hand-crafted features.

**Results:**

We present a recurrent neural network (RNN) framework based on word embeddings and character representation. On top of the neural network architecture, we use a CRF layer to jointly decode labels for the whole sentence. In our approach, contextual information from both directions and long-range dependencies in the sequence, which is useful for this task, can be well modeled by bidirectional variation and long short-term memory (LSTM) unit, respectively. Although our models use word embeddings and character embeddings as the only features, the bidirectional LSTM-RNN (BLSTM-RNN) model achieves state-of-the-art performance — 86.55% F1 on BioCreative II gene mention (GM) corpus and 73.79% F1 on JNLPBA 2004 corpus.

**Conclusions:**

Our neural network architecture can be successfully used for BNER without any manual feature engineering. Experimental results show that domain-specific pre-trained word embeddings and character-level representation can improve the performance of the LSTM-RNN models. On the GM corpus, we achieve comparable performance compared with other systems using complex hand-crafted features. Considering the JNLPBA corpus, our model achieves the best results, outperforming the previously top performing systems. The source code of our method is freely available under GPL at https://github.com/lvchen1989/BNER.

## Background

With the explosive increase of biomedical texts, information extraction, which aims to unlock structured information from raw text, has received more and more attention in recent years. Biomedical named entity recognition (BNER), which recognizes important biomedical entities (e.g. genes and proteins) from text, is a essential step in biomedical information extraction.

Because BNER is a fundamental task, it becomes the focus of some shared-task challenges, such as BioCreative II gene mention (GM) task [1] and JNLPBA 2004 task [2]. Most systems employed machine learning algorithms in BNER, likely due to the availability of the annotated datasets and promising results. Various machine learning models have been used for this task, such as Conditional Random Fields (CRFs) [3–7], Support Vector Machines (SVMs) [8], Maximum Entropy Markov Model (MEMM) [9] and Hidden Markov Model (HMM) [10]. These machine learning algorithms use different kinds of features, including orthographic, morphological, part-of-speech(POS) and syntactic features of words, word cluster features and domain-specific features using external resources, such as BioThesaurus [11]. However, the success of these approaches heavily depends on the appropriate feature set, which often requires much manual feature engineering effort for each task.

The rapid development of deep learning on many tasks (e.g., [12–15]) brings hope for possibly alleviating the problem of avoiding manual feature engineering. It provides a different approach that automatically learns latent features as distributed dense vectors. Recurrent neural network (RNN) [16] and its variants long-short term memory (LSTM) [17] have been successfully used in various sequence prediction problems, such as general domain NER [18, 19], language modeling [20, 21] and speech recognition [22].

Meanwhile, recent advances in word embedding induction methods [12, 23–25] have benefited researchers in two ways: (1) Intuitively, word embeddings can be used as extra word features in existing natural language processing (NLP) systems, including the general domain [26] and biomedical domain [27, 28], to improve the performance, and (2) they have enabled more effective training of RNNs by representing words with low dimensional dense vectors. which can capture distributional syntactic and semantic information [29, 30].

In this paper, we propose a neural network architecture for BNER. Without any external resources or hand-crafted features, our neural network method can be successfully used for this task. To capture morphological and orthographic information of words, we first use an attention model to encode character information of a word into its character-level representation. Then we combine character- and word-level representations and then feed them into the LSTM-RNN layer to model context information of each word. On top of the neural network architecture, we use a CRF layer to jointly decode labels for the whole sentence. Several word embeddings trained from different external sources are used in our LSTM-RNN models.

We evaluate our model on two BNER shared tasks — BioCreative II GM task and JNLPBA 2004 task. Experimental results on both corpus show that domain-specific pre-trained word embeddings and character-level representation can improve the performance of the LSTM-RNN models. Although our models use character embeddings and word embeddings as the only features, the bidirectional LSTM-RNN(BLSTM-RNN) model achieves state-of-the-art performance on both corpora.

## Methods

We regard BNER as a sequence labeling problem following previous work. The commonly used BIEOS tagging schema (B-beginning, I-inside, E-end, O-outside and S-the single word entity) is used to identify the boundary information of the entities.

### Overall architecture

**Fig. 1:**
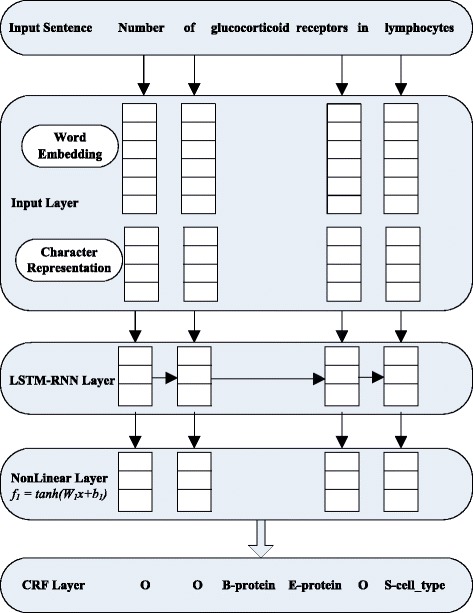
The model architecture

Figure 1 illustrates the overall architecture of our approach.

The input layer calculates the representation of input words based on both word and character embeddings. An attention model is used to compute the character-level representation of the word with the character embeddings as inputs. Then we combine the character representation and word embedding to get the feature representation of each word in the sentence.

The extracted features of each word are then passed through non-linear LSTM-RNN hidden layer, which is designed to combine the local and contextual information of a word. The forward LSTM and the backward LSTM can also be integrated into this layer. A nonlinear hidden layer *f*_1_ follows to form more complex features automatically.

Finally, the output vectors of the neural network are fed into a CRF layer. For a given input sentence, we model the label sequence jointly using the CRF, which considers the correlations between labels in neighborhoods.

### Input layer

Given an input sentence *s* as an ordered list of *m* words { *w*_1_, *w*_2_... *w*_*m*_}, the input representation $\vec x$ of the LSTM-RNN layers is computed based on both word and character embeddings.

To obtain the character representation of the word *w*_*i*_, we denote the character sequence of *w*_*i*_ with { *c*_1_, *c*_2_... *c*_*n*_}, where *c*_*j*_ is the *j*th character. The character embedding lookup table function $\vec e_{c}$ is used to map each character *c*_*j*_ into its character embedding $\vec e_{c}^{j}$. Then we use an attention model [31] to combine the character embeddings {$\vec e_{c}^{1}$, $\vec e_{c}^{2}$... $\vec e_{c}^{n}$} for *w*_*i*_. In this model, $\vec R^{i}_{c} = \sum _{j=1}^{n}a^{j}_{c}\odot \vec e_{c}^{j}$, where $\vec R^{i}_{c}$ is the character representation of *w*_*i*_, $a^{j}_{c}$ is the weight for $\vec e_{c}^{j}$, ⊙ is the Hadamard product function and $\sum _{j=1}^{n}a^{j}_{c}=1$.

Each $a^{j}_{c}$ is computed based on both the word embedding of the current word *w*_*i*_ and the character embedding window around the current character $\vec e_{c}^{j}$.

1 $$ \vec h^{j}_{c} = tan\left(W_{c}\left(\vec e_{c}^{j-2} \oplus \vec e_{c}^{j-1} \oplus \vec e_{c}^{j} \oplus \vec e_{c}^{j+1} \oplus \vec e_{c}^{j+2}\right)+b_{c}\right)  $$

2 $$ t^{j}_{c} = exp\left(W_{t}\vec h^{j}_{c}+U_{t}\vec e_{w}^{i}+b_{t}\right)  $$

3 $$ a^{j}_{c} = \frac{t^{j}_{c}}{\sum_{j=1}^{n}t^{j}_{c}}  $$

where ⊕ is the vector concatenation function and $\vec e_{w}^{i}$ is the embedding of the current word *w*_*i*_. *W*_*c*_,*W*_*t*_,*U*_*t*_,*b*_*c*_ and *b*_*t*_ are mode parameters. We combine the character representation $\vec R^{i}_{c}$ and word embedding $\vec e_{w}^{i}$ to form the representation $\vec R^{i}$: $\vec R^{i} = \vec R^{i}_{c} \oplus \vec e_{w}^{i}$.

Finally, the input representation $\vec x$ of the LSTM-RNN layer is computed by a window function: $\vec x_{i} = \vec R_{i-2} \oplus \vec R_{i-1} \oplus \vec R_{i} \oplus \vec R_{i+1} \oplus \vec R_{i+2}$.

### Long short-term memory RNN

The RNNs in this section are neural networks, which have recurrent connections and allow a form of memory. This makes them captures information about what has been calculated so far. They compute compositional vector representations for the input word sequences. These distributed representations are then used as features to predict the label of each token in the sentence.

Although RNNs can, in principle, model long-range dependencies, training them is difficult in practice, likely due to the vanishing and exploding gradient problem [32].

In this paper, we apply Long Short-Term Memory (LSTM) [17] to this task. LSTMs are variants of the above RNNs, with the recurrent hidden layer updates in RNNs are replaced with the special memory units. They have been shown to be better at capturing long range dependencies in the sequence data.

### Bidirectionality

With the definition of LSTM described above, we can see that the hidden state at time *t* only captures information from the past. However, both past (left) and future (right) information could also be beneficial for our task. In the sentence “Characterization of thyroid hormone receptors in human IM-9 lymphocytes.”, it helps to tag the word “thyroid” as **B-protein**, if the LSTMs know the following word is “receptors”.

To incorporate the future and past information, we extend LSTM with bidirectional approach, referred as the bidirectional LSTM [33], which allow bidirectional links in the network. Two separate hidden states $\overrightarrow {h_{t}}$ and $\overleftarrow {h_{t}}$ are used to represent the forward and backward sequence respectively. Finally, we combine the features from the forward and backward LSTMs by an hidden layer *f*_1_. The final output hidden layer *h*_*t*_ is computed as follows:

4 $$ h_{t}= tanh\left(W_{f}\left[\overrightarrow{h_{t}}; \overleftarrow{h_{t}}\right]+b_{f}\right)  $$

where $\overrightarrow {h_{t}}$ is the forward LSTM layer and $\overleftarrow {h_{t}}$ is the backward LSTM layer. *W*_*f*_ and *b*_*f*_ denote the weight matrix and bias vector in the hidden layer *f*_1_. The output feature representation *h*_*t*_ is then fed into the CRF layer and captures both the future and past information.

### CRF

For sequence labeling (or general structured prediction) tasks, it is beneficial to consider the correlations between labels in neighborhoods, and jointly decode the best chain of labels for a given input sentence. We model label sequence jointly using a CRF [34], instead of decoding each label independently.

For an input sentence **x**=*x*_1_,…,*x*_*T*_, the corresponding hidden sequence **h**=*h*_1_,…,*h*_*T*_ is output by the above neural networks. We consider the matrix *F* of scores $f_{\theta }\left ([h]^{T}_{1}\right)$ and *θ* is a model parameter of the CRFs. In the matrix *F*, the element *f*_*i,t*_ represents the score for the *t*-th word with the *i*-th tag. We introduce a transition score [*A*]_*j,k*_, which is also a model parameter, to model the transition from the *j*-th tag to the *k*-th tag. The score of the sentence $[x]^{T}_{1}$ along with a label sequence $[y]^{T}_{1}$ is computed by summing the transition scores and network output scores:

5 $$ S\left([x]^{T}_{1};[y]^{T}_{1}\right) = \sum_{t=1}^{T}(A_{y_{t-1}, y_{t}} + f_{y_{t}, t})  $$

Then given the sentence **x**, the conditional probability of a label sequence **y** is defined by the following form:

6 $$ P(\mathbf{y}|\mathbf{x}) = \frac{exp{S\left([x]^{T}_{1};[y]^{T}_{1}\right)}}{\sum_{y'\in Y(x)}exp{S\left([x]^{T}_{1};[y']^{T}_{1}\right)}}  $$

where *Y*(*x*) denotes all the possible label sequences for the sentence **x**.

The label sequence $\widehat {y}\in Y(x)$ with the highest score is the predicted sequence for sentence **x**:

7 $$ \widehat{y} = \underset{y \in Y(x)}{\text{argmax}} P(\mathbf{y}|\mathbf{x})  $$

For the CRF model, decoding can be solved efficiently by adopting the Viterbi algorithm.

### Training

Max likelihood objective are used to train our model. The parameters *Θ* is the parameter set in our model. It consists of the parameters *W* and *b* of each neural layer, and the model parameters in the CRF layer.

Given the training examples set $\mathcal {B}$, the log-likelihood objective function is defined as:

8 $$ L(\Theta) = \frac{1}{|\mathcal{B}|}\sum_{(x_{n},y_{n}) \in \mathcal{B}}logP(y_{n}|x_{n}) +\frac{\lambda}{2} \parallel \Theta \parallel^{2}  $$

where *logP*(*y*_*n*_|*x*_*n*_) is the log probability of *y*_*n*_ and *λ* is a regularization parameter.

To maximum the objective, we use online learning to train our model, and the AdaGrad algorithm [35] is used to update the model parameters. The parameter update at time *t* for the *j*-th parameter *θ*_*j,t*_ is defined as follows:

9 $$ \theta_{j,t} = \theta_{j,t-1} - \frac{\alpha}{\sqrt{\sum_{\tau =1}^{t}g_{j,\tau}^{2}}}g_{j,t}  $$

where *α* is the initial learning rate, and *g*_*j*,*τ*_ is the subgradient for the *j*-th parameter at time *τ*.

### Word embedding

Word embeddings are distributed representations and capture distributional syntactic and semantic information of the word. Several types of word embeddings trained from different external sources are used in our LSTM-RNN models. Here we will give a brief description of these pre-trained word embeddings.

#### SENNA

Collobert et al. (2011) [12] propose a neural network framework for various NLP tasks. To give their network a better initialization, they introduce a new neural network model to compute the word embeddings. The main idea for the neural network is to output high scores for positive examples and low scores for negative examples. The positives example are the word windows in a large unlabeled corpus, and the negative examples are the windows where one word is replaced by a random word.

They releases the word embeddings with the 130K vocabulary words [36]. The dimension of the SENNA word embedding is 50 and they are trained for about 2 months, over English Wikipedia.

#### Word2vec

Another start-of the-art method word2vec [23, 24] can be used to learn word embeddings from large corpus efficiently. They propose the continuous bag-of-words (CBOW)model and the skip-gram model for computing word embeddings.

They release pre-trained vectors with 3 million vocabulary words. The dimension of the word2vec word embeddings is 300 and the training corpus is part of Google News dataset [37].

#### Biomedical embeddings

Since we work on biomedical text, which is different from the above general domain corpora, domain-specific embeddings are trained using the word2vec CBOW model from a set of unannotated data. The corpus contains all full-text documents from the PubMed Central Open Access subset [38].

For comparison with SENNA and Google word2vec embeddings, we learn word embeddings (vocabulary size 5.86 million) of 50- and 300-dimensions using the word2vec tool [23, 24].

## Results and discussion

### Data sets

We evaluate our neural network model on two publicly available corpora: the BioCreAtIvE II GM corpus and JNLPBA corpus, for system comparison with existing BNER tools. The GM corpus consists of 20,000 sentences (15,000 sentences for training and 5000 sentences for test) from MEDLINE, where gene/gene product names(grouped into only one semantic type) were manually annotated. On the other hand, the JNLPBA corpus consists of 22,402 sentences (18,546 training sentences and 3856 test sentences) from MEDLINE abstracts. The manual annotated entities in JNLPBA corpus contains five types, namely DNA, RNA, protein, cell line, and cell type. In addition,10% of the training set are randomly split as the development data to tune hyper-parameters during training. Table 1 shows the statistics of the two corpora.

**Table 1:** Statistics of the datasets

	Training	Dev	Test
GM			
Sentences	13500	1500	5000
One-word Entities	7051	805	2831
Multi-word Entities	9355	1047	3494
Total Entities	16406	1852	6325
JNLPBA			
Sentences	16691	1855	3856
One-word Entities	19476	2170	3466
Multi-word Entities	26765	2890	5196
Total Entities	46241	5060	8662

### Evaluation metric

We evaluate the results in the same way as the two shared tasks, using precision (P), recall (R) and F1 score (F1):

10 $$ P = \frac{TP}{TP+FP}  $$

11 $$ R = \frac{TP}{TP+FN}  $$

12 $$ {}F1 = \frac{2 \times P \times R}{P + R}  $$

where *TP* is the number of correct spans that the system returns, *FP* is the number of incorrect spans that the system returns, and *FN* is the number of missing spans.

Note that alternative annotations generated by human annotators in the GM corpus will also count as true positives. We evaluate the result on the GM coups using the official evaluation script.

### Neural network settings

#### Pre-processing

We transform each number with *NUM* and lowercase all words in the pre-process step. We also mark the words, which are not in the word embedding vocabulary, as *UNKNOWN*.

#### Parameters

Character embeddings are randomly initialized with uniform samples from range [0,1] and we set the dimension of character embeddings to 30.

For each neural layer in our neural network model, parameters *W* and *b* are randomly initialized with uniform samples from $[-\sqrt {\frac {6}{nr+nc}}$, $+\sqrt {\frac {6}{nr+nc}}]$, where *nr* and *nc* are the number of rows and columns of *W*. The initial learning rate for AdaGrad is 0.01 and the regularization parameter is set to 10^−8^.

The dimension of the single RNN hidden layer *h*_1_ is 100 and the size of hidden layers *f*_1_ connected to RNN hidden layer *h*_2_ is set to be 100. Tuning the hidden layer sizes can not significantly impact the performance of our model.

#### Code

The C++ implementations of our proposed models are based on the LibN3L package [39], which is a deep learning toolkit in C++.

### Experimental results

Table 2 presents our results on BioCreative II GM and JNLPBA data sets for various LSTM-RNNs and word embeddings.

**Table 2:** Results for various LSTM-RNNs and word embeddings on the GM and JNLPBA data sets

Systems	Dim.	GM (P/R/F1 score)	JNLPBA (P/R/F1 score)
LSTM-RNN			
+SENNA	50	83.87/80.46/82.13	67.50/72.52/69.92
+Biomedical	50	85.85/84.09/84.96	70.69/74.80/72.69
+Google	300	83.90/82.80/83.35	69.19/72.56/70.83
+Biomedical	300	86.66/85.58/86.12	70.34/74.96/72.58
+Random	300	83.63/76.56/79.94	66.96/71.46/69.13
BLSTM-RNN			
+SENNA	50	84.29/79.83/82.00	67.00/71.60/69.22
+Biomedical	50	88.42/82.63/85.43	71.04/74.45/72.71
+Google	300	85.02/82.04/83.50	68.59/73.99/71.19
+Biomedical	300	87.85/85.29/86.55	71.24/76.53/73.79
+Random	300	82.87/77.65/80.18	68.43/70.98/69.68

#### Contributions of word embeddings in LSTMs

In our LSTM framework, word embeddings are used to avoid feature engineering efforts, and these embeddings are not fine-tuned in the experiments above. Despite using these pretrained word embeddings, we can also randomly initialize the word embedding in the neural network.

To show the contributions of word embeddings, we perform experiments with different pretrained word embeddings, as well as a random initialization embeddings. According to the results in Table 2, models using pretrained word embeddings significantly performs better than the Random ones by providing better initialization, with the maximum gains of 6.37% on GM and 4.11% on JNLPBA by **BLSTM + Biomedical** (300 dim.). The results are significant at *p*<10^−3^ by pair-wise t-test.

For different pretrained embeddings, the domain-specific biomedical embeddings (300 dim.) achieve best results in all cases. For example, BLSTM-RNN using biomedical embeddings (300 dim.) outperforms the SENNA (50 dim.) ones, with the gain of 4.55% (*p*<10^−3^) on GM and 4.57% (*p*<10^−3^) on JNLPBA. The possible reasons are that:(1) it is trained on the biomedical domain corpus and (2) high dimensional embeddings may capture more information compared with the low dimensional ones. Biomedical embeddings (300 dim.) can capture more syntactic and semantic information, and improves the performance on this task.

#### Comparison between bidirectional and unidirectional

When we compare the uni-directional LSTM-RNNs with their bidirectional counterparts, we can see that the bidirectional improves the performance. BLSTM significantly outperforms LSTM with the maximum gains of 0.43% on GM and 1.21% on JNLPBA by **BLSTM-RNN + Biomedical** (300 dim.).

However, these improvements does not meet our expectation. When we analyze the data set, we find it to be unsurprising because of the span distribution in the data set. The average span of the named entity mentions in JNLPBA data set is two words, and 40.0% of the mentions only contain one word. Despite these named entity mentions, there are still 15.4% of the mentions whose span is more than 3. Therefore, the information captured by the bidirectional link helps to correctly recognize these mentions.

#### Effects of fine-tuning word embeddings

Table 3 shows the F1 score of LSTM-RNN and BLSTM-RNN, when the embeddings are not fine-tuned in the training process, and when they are learned as part of model parameters (fine-tuned) in the task.

**Table 3:** Effects of fine-tuning word embeddings in LSTM-RNN and BLSTM-RNN

Systems	Dim.	GM		JNLPBA	
LSTM-RNN		+tune	-tune	+tune	-tune
+SENNA	50	85.69	82.13	70.56	69.92
+Biomedical	50	85.33	84.96	71.78	72.69
+Google	300	85.65	83.35	71.13	70.83
+Biomedical	300	84.56	86.12	72.04	72.58
+Random	300	84.74	79.94	71.10	69.13
BLSTM-RNN		+tune	-tune	+tune	-tune
+SENNA	50	86.81	82.00	72.09	69.22
+Biomedical	50	85.24	85.43	72.28	72.71
+Google	300	86.52	83.50	73.03	71.19
+Biomedical	300	84.53	86.55	73.44	73.79
+Random	300	84.94	80.18	71.81	69.68

Considering SENNA, Google and Random embeddings, fine-tuning these three embeddings in our LSTM framework significantly outperforms the non-tuned settings, with a maximum absolute gain of 4.81% (*p*<10^−3^) on GM and 2.87% (*p*<10^−3^) on JNLPBA by **BLSTM + SENNA**. These embeddings are not good initialization for our neural model, and fine-tuning them in our LSTM framework can improve the performance on this task. The likely reason is that these embeddings are trained on general domain or randomly initialization, and may have much noise for this task. Remarkably, fine-tuning brings the performance of Random initialization close to the best ones.

Considering the domain-specific biomedical embeddings, using them without fine-tuning significantly performs better that the fine-tuned ones, with a maximum absolute gain of 2.02% (*p*<10^−3^) on GM by **BLSTM + Biomedical** (300 dim.). Fine-tuning the biomedical embeddings is not necessary in our model, and it may cause slight overfitting and reduce the performance.

#### Effects of character representation

**Fig. 2:**
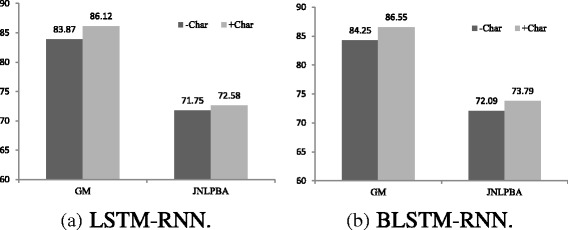
Effects of character representation. +Char — with character representation; -Char — without character representation. **a** LSTM-RNN, **b** BLSTM-RNN

Figure 2 shows the effects of character representation in our LSTM framework for each data set. Biomedical embeddings (300 dim.) are used in our experiments without fine-tuning.

From the Fig. 2, we observe an essential improvement on both data sets. Compared with the model without character representation, the model with character representation improves the F1 score with the gain of 2.3% on GM and 1.7% on JNLPBA by BLSTM. It demonstrates the effectiveness of character representation in BNER.

#### Effects of the CRFs

In this section, we conduct the experiments to show the effects of the CRF layer in our framework. Instead of the CRFs, *softmax* classfier can be also used to predict the label of each token based on the feature representation output by our LSTM framework. The *softmax* classfier layer calculates the probability distribution over all labels and chooses the label with highest probability for each word.

Table 4 shows the performance of BLSTM-RNN models with and without the CRF layer. Biomedical embeddings (300 dim.) are used in the experiments without fine-tuning. We can see that the CRF layer significantly (*p*<10^−3^) improves the performance with the gain of 3.91% on GM and 1.86% on JNLPBA.

**Table 4:** Comparison of systems with and without the CRF layer

Systems	GM	JNLPBA
BLSTM-RNN	82.64	71.93
BLSTM-RNN+CRF	86.55	73.79

The improvements show that although BLSTM is considered to have the ability of handing sequential data and can automatically model the context information, it is still not enough. And the CRF layer, which jointly decode label sequences, helps to benefit the performance of the LSTM models in BNER.

#### Feature representation plotting

Although neural networks have been successfully used for many NLP tasks, the feature representation of the NN models is difficult to understand. Inspired by the work of Li et al. (2016) [40], which visualizes and understands phrase/sentence representation for sentiment analysis and text generation, we conduct the experiment to visualize the feature representation in our LSTM models for BNER.

**Fig. 3:**
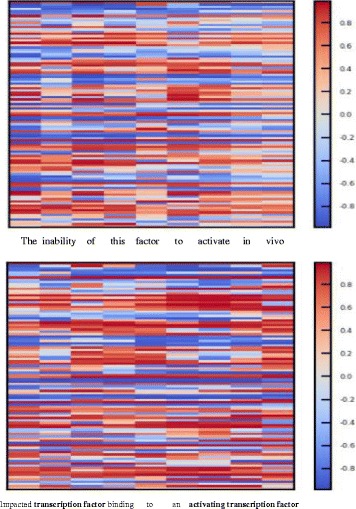
Feature representation of our model. Each column indicates the feature representation from BLSTM for each token. Each grid in the column indicates each dimension of the feature representation. The dimension of the feature representation is 100

Figure 3 shows the heat map of the feature representations of some context in two sentences. The representations are the input features of the CRF layer in our framework. The **BLSTM + Biomedical** (300 dim.) model is used in the experiments.

These two cases are namely “the inability of this factor to activate in vivo the...” and “In particular, naturally occurring sequence variation impacted *transcription factor* binding to an *activating transcription factor* / cAMP response element...”. In the first context, the word “factor” occurs in a general noun phrase without any descriptive words and is not identified as an entity. While in the second context, two entity mentions, namely “transcription factor” and “activating transcription factor”, are recognized with the Protein type. The representation of the word “factor” in the first sentence is different from the entity mentions in the second sentence.

**Fig. 4:**
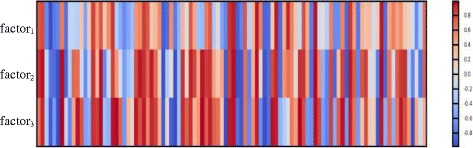
Feature representation of the word “factor”. “factor_1_” is the word in the first sentence. “factor_2_” and “factor_3_” are the corresponding words in the second sentence. Each vertical bar indicates one dimension of the feature representation for the corresponding word

In particular, Fig. 4 shows the heat map of the feature representation of the word “factor”. Our LSTM model outputs different representation for it in different context. We can see that the representation difference between the word “factor_1_” and the other two words “factor_2_” and “factor_3_” is apparent. While the representation of the words “factor_2_” and “factor_3_”, both recognized as part of entities, are similar.

This is an initial experiment for understanding the ability of our feature representation to predict the label in BNER task. More strategies for understanding and visualizing neural models need to be explored in future work.

#### Comparison with previous systems

Tables 5 and 6 illustrate the results of our model on the GM and JNLPBA corpus respectively, together with previous top performance systems for comparison. IBM [41] and Infocomm [8] are the best systems participating in BioCreative II GM task and JNLPBA task respectively.

**Table 5:** Results of our model on the GM corpus, together with top-performance systems

Systems	P/R/F1
BLSTM + Biomedical (300 dim.)	**87.85/85.29/86.55**
AIIAGMT [5]	**88.95/87.65/88.30**
IBM [41]	88.48/85.97/87.21
Gimli [3]	90.22/84.82/87.17
BANNER [6]	88.66/84.32/86.43
NERSuite [4]	88.81/82.34/85.45
Li et al. (2015) [30]	83.29/80.50/81.87
NERBio [7]	92.67/68.91/79.05

**Table 6:** Results of our model on the JNLPBA corpus, together with top-performance systems

Systems	P/R/F1
BLSTM + Biomedical (300 dim.)	**71.24/76.53/73.79**
NERBio [7]	**72.01/73.98/72.98**
Infocomm [8]	69.42/75.99/72.55
Gimli [3]	72.85/71.62/72.23
NERSuite [4]	69.95/72.41/71.16

IBM [41] uses semi-supervised machine learning method and forward and backward model combination, while Infocomm [8] combines HMM and SVM model to tackle this task. CRFs are widely used in BNER-shared tasks and have shown the state-of-the-art performance [3–7]. The performance of these systems depends on manually extracted rich features.

Note that these systems use complex features like orthographic, morphological, linguistic features and many more in their models, some of which rely on external resources. In addition, some systems also use model combination strategy and integrate post-processing modules, including abbreviation resolution and parentheses correction. Our LSTM-RNNs only use character representation and word embeddings as input features, avoiding manual feature engineering.

In recent years, deep neural network architectures have been proposed and successfully applied to BNER. Li et al. (2015) [30] applies extended Elman-type RNN to this task and the results on BioCreative II GM data set show that extended RNN outperforms CRF, deep neural networks and original RNN.

On the GM corpus, our model achieves 4.68% improvements of F1 score over Li et al. (2015) [30], which is a neural network model using used softmax function to predict which tag the current token belongs to. This demonstrates the effectiveness of our Bi-LSTM-CRF for this task and the importance of character representation. Comparing with traditional statistical models, our best model **BLSTM+Biomedical** (300 dim.) gives competitive results on F1 score. Considering the JNLPBA corpus, our best model **BLSTM+Biomedical** outperforms all these previous systems, with a significant improvement of 0.81% over the NERBio system.

#### Error analysis

For BNER task, the errors contain two categories, including false positives (FP) and false negatives (FN). The entities in JNLPBA corpus contain five types, while the entities in GM corpus are grouped into only one type. We analyze the errors on the JNLPBA test set and report the results in this section.

Both FP and FN errors can be further divided into two types: 1) Boundary errors, in which the boundary of an entity is incorrectly identified. 2) Type errors, in which the boundary of an entity is correct but its type is incorrectly identified. Table 7 shows the statistics of error analysis. The boundary errors are the main errors and constitute more than 80% of all errors in both FP and FN errors.

**Table 7:** Error analysis on JNLPBA test set

Error type	%	
FP	Boundary errors	49.31
	Type errors	7.52
FN	Boundary errors	35.66
	Type errors	7.52

We further distinguish these errors into the following categories:

- 1) Left boundary errors. These errors are recognized with wrong left boundary and correct right boundary. The recognized entities often include too many details (“cytosol estradiol receptors” rather than just “estradiol receptors”) or too few (“B lineage genes” instead of “many B lineage genes”). In these errors, some words, such as “factor” and “protein”, are very useful indictors for entity mentions with the type Protein. While some words, such as “genes” and “sites”, are useful for entity recognition with the type DNA. The right boundary is correctly recognized in these cases and it is difficult for us to determine whether the descriptive words are parts of the entity mentions (e.g. “normal” in “normal human lymphocytes”).
- 2) Coordinate entity errors. The coordinate entity names, such as “*upstream promoter* or *enhancer element*” and “*NF-kappa B* and *AP-1 binding sites*”, are often combined with some coordinating conjunctions. It is difficult to distinguish whether they are one whole entity or not. For example, “upstream promoter or enhancer element [DNA]” is identified as two entities “upstream promoter [DNA]” and “enhancer element [DNA]” by our system. There are also some apposition entities, such as “transcription factor NF-kappa B” and they are frequently recognized as two individual entities (e.g. “transcription factor [Protein]” and “NF-kappa B [Protein]” instead of “transcription factor NF-kappa B [Protein]”) by our system.
  This may be rational due to the following reasons: First, the components of the entities are frequently annotated as an individual entity when they occur alone in the corpus. For example, both “transcription factor” and “NF-kappa B” are often annotated with the type Protein. Second, these errors are mainly caused by the corpus annotation inconsistency. The above coordinate entities are annotated as one whole entity in some sentences. While in other sentences, these entity mentions are annotated as multiple individual entities.
- 3) Missing entities. They include the annotated entities, which are not matched (or overlapped) with any recognized entities. We find that 49.1% of these errors come from the Protein type and 48.3% of them are one word entities on the JNLPBA corpus. Among these errors, some general noun words (e.g. “antibodies” and “receptors”) are annotated as biomedical entities. In addition, abbreviations, such as “EZH2” and “IL-5”, can not be recognized by our model in some context.
  The missing entities on the JNLPBA data occur with a similar percentage on the GM data set. These errors are involved in 8.51% of all the entities on the JNLPBA corpus, while the percentage of the missing entities on the GM corpus is 9.72%. As to the single word entities, the percentage of them in the missing errors is 48.3% on the JNLPBA corpus, while the percentage of them on the GM corpus is 54.6%. The likely reason for the similar percentage is that Protein is the main type on the JNLPBA data and 58.5% of the entities come from the Protein type.
  The character representation helps to improve the model for the single word entities. When removing the character representation from our model, the percentage of the single word entities in the missing errors will increase from 48.3 to 56.4% on the JNLPBA corpus. In the future, more contextual information should be considered to improve the BNER.
- 4) Classification errors. They include the errors with correct boundary match but wrong type identification. We find that 35.6% of the errors are caused by misclassification of the Cell_type type to the Cell_line type and 31.5% of the errors are the misclassification of the DNA type to the Protein type, e.g. “IRF1 [Protein]” instead of “IRF1 [DNA]”. It is difficult to distinguish them, because of the sense ambiguity of these biomedical named entities.

From the above analysis, we find that some errors on the JNLPBA data are caused by the corpus annotation inconsistency. Considering the GM data, the F1 score of our model increases from 77.5 to 86.6% with the alternative annotations. Although our model achieves state-of-the-art performance on the JNLPBA corpus, more contextual information and external knowledge should be considered to improve the BNER.

## Conclusions

In this paper, we present a neural network architecture for this task. Our model can be successfully used for BNER task without any feature engineering effort.

In order to evaluate our neural network model and compare it to other existing BNER systems, we use two commonly used corpora: GM and JNLPBA. Our best model **BLSTM+Biomedical** (300 dim.) model achieves F1 score results of 86.55% and 73.79% on each corpus, respectively. Experimental results on both corpora demonstrate that pre-trained word embeddings and character representation both improve the performance of the LSTM-RNN models. Although our models use word embeddings and character embeddings as the only features, we achieve comparable performance on the GM corpus, comparing with other systems using complex hand-crafted features. Considering the JNLPBA corpus, our model achieves the best results, outperforming these previously top performing systems.

In the future, we will explore the effects of adding depth to the LSTM layers. In this paper, our LSTM framework only contains one LSTM hidden layer. We can design multiple LSTM hidden layers and higher LSTM layers may help to exploit more effective features in deeper networks. Another direction is that we plan to apply our method to other related tasks, such as biomedical relation extraction. We would also like to explore to jointly model these tasks in the RNN-based framework.

## Abbreviations

BLSTM: Bidirectional long short-term memory
BNER: Biomedical named entity recognition
CRFs: Conditional random fields
FN: False negative
FP: False positive
GM: Gene mention
HMM: Hidden Markov Model
LSTM: Long short-term memory
MEMM: Maximum Entropy Markov Model
NLP: Natural language processing
P: Precision
R: Recall
RNN: Recurrent neural network
SVMs: Support vector machines

## References

[1] Smith L, Tanabe LK, nee Ando RJ, Kuo CJ, Chung IF, Hsu CN, Lin YS, Klinger R, Friedrich CM, Ganchev K (2008). Overview of biocreative ii gene mention recognition. Genome Biol.

[2] Kim JD, Ohta T, Tsuruoka Y, Tateisi Y, Collier N (2004). Introduction to the bio-entity recognition task at jnlpba. Proceedings of the International Joint Workshop on Natural Language Processing in Biomedicine and Its Applications.

[3] Campos D, Matos S, Oliveira JL (2013). Gimli: open source and high-performance biomedical name recognition. BMC Bioinformatics.

[4] Cho H, Okazaki N, Miwa M, Tsujii J. Nersuite: a named entity recognition toolkit. Tsujii Laboratory, Department of Information Science, University of Tokyo, Tokyo, Japan [http://nersuite.nlplab.org/index.html]. 2010.

[5] Hsu CN, Chang YM, Kuo CJ, Lin YS, Huang HS, Chung IF (2008). Integrating high dimensional bi-directional parsing models for gene mention tagging. Bioinformatics.

[6] Leaman R, Gonzalez G (2008). Banner: an executable survey of advances in biomedical named entity recognition. Pacific Symposium on Biocomputing, vol. 13.

[7] Tsai RT-H, Sung CL, Dai HJ, Hung HC, Sung TY, Hsu WL (2006). Nerbio: using selected word conjunctions, term normalization, and global patterns to improve biomedical named entity recognition. BMC Bioinformatics.

[8] GuoDong Z, Jian S (2004). Exploring deep knowledge resources in biomedical name recognition. Proceedings of the International Joint Workshop on Natural Language Processing in Biomedicine and Its Applications.

[9] Finkel J, Dingare S, Nguyen H, Nissim M, Manning C, Sinclair G (2004). Exploiting context for biomedical entity recognition: from syntax to the web. Proceedings of the International Joint Workshop on Natural Language Processing in Biomedicine and Its Applications.

[10] Zhao S (2004). Named entity recognition in biomedical texts using an hmm model. Proceedings of the International Joint Workshop on Natural Language Processing in Biomedicine and Its Applications.

[11] Liu H, Hu ZZ, Zhang J, Wu C (2006). Biothesaurus: a web-based thesaurus of protein and gene names. Bioinformatics.

[12] Collobert R, Weston J, Bottou L, Karlen M, Kavukcuoglu K, Kuksa P (2011). Natural language processing (almost) from scratch. J Mach Learn Res.

[13] Lyu C, Lu Y, Ji D, Chen B. Deep learning for textual entailment recognition. In: Proceddings of ICTAI 2015: 2015. p. 154–61. doi:10.1109/ICTAI.2015.35.

[14] Zeng T, Li R, Mukkamala R, Ye J, Ji S (2015). Deep convolutional neural networks for annotating gene expression patterns in the mouse brain. BMC Bioinformatics.

[15] Zhang M, Zhang Y, Vo DT (2016). Gated neural networks for targeted sentiment analysis. Proceedings of the Thirtieth AAAI Conference on Artificial Intelligence, Phoenix, Arizona, USA. Association for the Advancement of Artificial Intelligence.

[16] Elman JL (1990). Finding structure in time. Cogn Sci.

[17] Hochreiter S, Schmidhuber J (1997). Long short-term memory. Neural Comput.

[18] Huang Z, Xu W, Yu K. Bidirectional lstm-crf models for sequence tagging. 2015. arXiv preprint arXiv:1508.01991.

[19] Chiu JP, Nichols E (2016). Named entity recognition with bidirectional lstm-cnns. Trans Assoc Comput Linguist.

[20] Mikolov T, Karafiát M, Burget L, Cernocký J, Khudanpur S (2010). Recurrent neural network based language model. INTERSPEECH 2010.

[21] Sundermeyer M, Schlüter R, Ney H (2012). LSTM neural networks for language modeling. INTERSPEECH 2012.

[22] Graves A, Jaitly N (2014). Towards end-to-end speech recognition with recurrent neural networks. Proceedings of ICML 2014.

[23] Mikolov T, Chen K, Corrado G, Dean J. Efficient estimation of word representations in vector space. 2013. arXiv preprint arXiv:1301.3781.

[24] Mikolov T, Sutskever I, Chen K, Corrado GS, Dean J (2013). Distributed representations of words and phrases and their compositionality. Advances in Neural Information Processing Systems.

[25] Jiang Z, Li L, Huang D, Jin L (2015). Training word embeddings for deep learning in biomedical text mining tasks. Proceedings of BIBM 2015.

[26] Turian JP, Ratinov L, Bengio Y. Word representations: A simple and general method for semi-supervised learning. In: Proceedings of ACL 2010: 2010. p. 384–94.

[27] Tang B, Cao H, Wang X, Chen Q, Xu H. Evaluating word representation features in biomedical named entity recognition tasks. BioMed Res Int. 2014; 2014. doi:10.1155/2014/240403. 10.1155/2014/240403 PMC3963372 24729964

[28] Lu Y, Ji D, Yao X, Wei X, Liang X (2015). CHEMDNER system with mixed conditional random fields and multi-scale word clustering. J Cheminformatics.

[29] Irsoy O, Cardie C (2014). Opinion mining with deep recurrent neural networks. Proceedings of EMNLP 2014.

[30] Li L, Jin L, Jiang Z, Song D, Huang D. Biomedical named entity recognition based on extended recurrent neural networks. In: Proceedings of BIBM 2015: 2015. p. 649–52. doi:10.1109/BIBM.2015.7359761.

[31] Bahdanau D, Cho K, Bengio Y. Neural machine translation by jointly learning to align and translate. In: Proceedings of ICLR: 2015.

[32] Bengio Y, Simard P, Frasconi P (1994). Learning long-term dependencies with gradient descent is difficult. IEEE Trans Neural Netw.

[33] Graves A, Mohamed A-r, Hinton G (2013). Speech recognition with deep recurrent neural networks. 2013 IEEE International Conference on Acoustics, Speech and Signal Processing.

[34] Lafferty JD, McCallum A, Pereira FCN (2001). Conditional random fields: Probabilistic models for segmenting and labeling sequence data. Proceedings of ICML 2001.

[35] Duchi J, Hazan E, Singer Y (2011). Adaptive subgradient methods for online learning and stochastic optimization. J Mach Learn Res.

[36] Collobert R. SENNA. http://ronan.collobert.com/senna/. Accessed 5 Apr 2016.

[37] Mikolov T. word2vec. https://code.google.com/archive/p/word2vec/. Accessed 5 Apr 2016.

[38] PubMed Central Open Access Subset. https://www.ncbi.nlm.nih.gov/pmc/tools/openftlist/. Accessed 5 Aug 2016.

[39] Zhang M, Yang J, Teng Z, Zhang Y (2016). Libn3l: A lightweight package for neural NLP. Proceedings of the Tenth International Conference on Language Resources and Evaluation LREC.

[40] Li J, Chen X, Hovy EH, Jurafsky D. Visualizing and understanding neural models in NLP. In: NAACL HLT 2016, The 2016 Conference of the North American Chapter of the Association for Computational Linguistics: Human Language Technologies, San Diego California, USA, June 12-17, 2016: 2016. p. 681–91.

[41] Ando RK (2007). Biocreative ii gene mention tagging system at ibm watson. Proceedings of the Second BioCreative Challenge Evaluation Workshop, vol. 23.

